# Functional constraints on tooth morphology in carnivorous mammals

**DOI:** 10.1186/1471-2148-12-146

**Published:** 2012-08-16

**Authors:** Peter D Smits, Alistair R Evans

**Affiliations:** 1 Department of Biological Sciences, Monash University, Melbourne, VIC, 3800, AUS

**Keywords:** Three-dimensional reconstruction, Carnivora, Dasyuromorphia, Ecomorphology, Integration

## Abstract

**Background:**

The range of potential morphologies resulting from evolution is limited by complex interacting processes, ranging from development to function. Quantifying these interactions is important for understanding adaptation and convergent evolution. Using three-dimensional reconstructions of carnivoran and dasyuromorph tooth rows, we compared statistical models of the relationship between tooth row shape and the opposing tooth row, a static feature, as well as measures of mandibular motion during chewing (occlusion), which are kinetic features. This is a new approach to quantifying functional integration because we use measures of movement and displacement, such as the amount the mandible translates laterally during occlusion, as opposed to conventional morphological measures, such as mandible length and geometric landmarks. By sampling two distantly related groups of ecologically similar mammals, we study carnivorous mammals in general rather than a specific group of mammals.

**Results:**

Statistical model comparisons demonstrate that the best performing models always include some measure of mandibular motion, indicating that functional and statistical models of tooth shape as purely a function of the opposing tooth row are too simple and that increased model complexity provides a better understanding of tooth form. The predictors of the best performing models always included the opposing tooth row shape and a relative linear measure of mandibular motion.

**Conclusions:**

Our results provide quantitative support of long-standing hypotheses of tooth row shape as being influenced by mandibular motion in addition to the opposing tooth row. Additionally, this study illustrates the utility and necessity of including kinetic features in analyses of morphological integration.

## Background

The evolution of morphology is limited by the complex interactions of various selection pressures and constraints, which can be extremely difficult to quantify [1]. In the context of functional processes, morphological constraints are due to interactions within and between different structures and both biotic and abiotic selection pressures [2-4]. A classical example is scaling in animals where traits such as femur diameter increase non-isometrically with mass because fundamental physical forces prevent certain morphologies from being adaptively feasible. Functional constraints on morphology are not limited to scaling, but may be caused by a wide range of interactions such eye structure and nocturnality [5], flight cost in relation to weather [6], shell shape and marine habitat [7,8], skull shape and bite force [9-11], among many others.

Morphological integration is both a cause and product of constraint and refers to how certain structures vary more closely with each other than with other structures because of various constraints [12,13], based on either *a priori* or *a posteriori* biological hypotheses [14]. Integration, in this sense, can be thought of as a biotic selection mechanism, where morphology is constrained by the complex interaction of many different features. Identifying strongly integrated features and the patterns of this integration has been a very active field of research [15] and systems ranging from insect wings [16,17] and mammal mandibles [18,19], to trilobites [20-22] have all been analyzed under various contexts. Methodologically, many studies have focused on static morphological features and defined patterns of integration based on correlation within and among structures. In this study, we use a combination of static and kinetic features to understand integration in carnivore teeth and mandibular movement. The combination of static and kinetic measures has not previously been used in studies of integration and provides a new and powerful approach to understanding functional morphology.

### Teeth

Opposing mammalian teeth interact in a very precise manner, with specific tooth features occluding with each other, and the molars of mammals have specialized shapes related to diet [23,24]. The complexity of the constraints on tooth morphology is not well understood. There has been extended speculation about the relationship between tooth row shape and mandibular movement. Ryder [25] suggested a relationship between occlusal shape and the relative amount of lateral translation of the mandible. Simpson [26] hypothesized that the amount of ventral and/or lateral movement plays an important role in controlling tooth shape. Here, we quantify whether the addition of measures of mandibular motion is necessary to better statistically model the effect of opposing tooth rows upon their shape. With the advent of three dimensional methods for analyzing morphology and application of model selection methods, it is possible to compare the integration of static and kinetic features and determine if a more complex hypothesis is appropriate for understanding tooth row shape.

**Figure 1:**
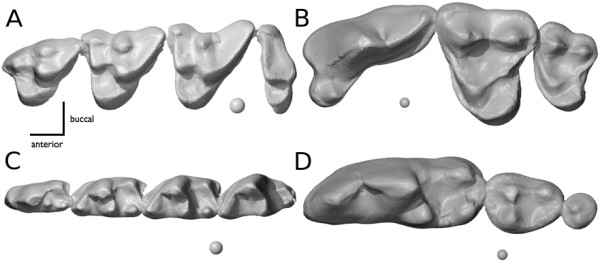
**Comparison of upper and lower tooth rows in Carnivora and Dasyuromorphia.** 3-dimensional renderings of upper tooth rows of *Vulpes vulpes* (**A**) and *Dasyurus geoffroii* (**B**) and lower tooth rows (**C, D**). Scale spheres are 1 mm in diameter.

Because Carnivora have a highly variable number of molars and Dasyuromorphia consistently have four molars (Figure 1), homology-based methods are inappropriate. Recently, an homology free measure of tooth row complexity has been developed [24] which allows for comparisons among tooth rows of varying length. This measure, called orientation patch count (OPC), is calculated from three-dimensional tooth surfaces. It is the sum of the number of orientation-delimited surfaces on the tooth row (Figure 1). Low OPC values correspond with faunivory while high OPC values with herbivory. OPC has been used to quantify variation in dental complexity for Carnivora and Rodentia [24], plesiadapids [27], primates [28], bats [29], and multituberculates [30].

While OPC is a quantification of the complexity of a tooth row, it is not a statement of the direction of occlusion for the tooth row and there has been no evidence to indicate that tooth row complexity is related to occlusal direction or mandible motion. While it is logical that opposing tooth row complexities of a taxon would be highly correlated [24] it is unknown whether some measure of mandibular motion in addition to the opposing tooth row shape allows for a more informative explanation of tooth row shape. We may expect that as tooth row complexity increases, jaw movement would need to change to ensure effective use of the change in tooth shape. Additionally, it is unknown if upper or lower tooth rows have different explanatory relationships with the opposing tooth row and mandibular motion. This uncertainty reveals multiple hypotheses which cannot be eliminated *a priori*.

Carnivora and Dasyuromorphia are good systems for comparing hypotheses about similarities in tooth shape and movement during occlusion because of ecological similarities, gross morphological differences and because mandibular movement is constrained to being only rotation and mediolateral translation with extremely limited anteroposterior movement. In this study, we compare biological hypotheses of tooth-mastication integration in carnivorous mammals. Statistical models of tooth row OPC as a response solely to opposing tooth row OPC may be the most parsimonious explanation of tooth row shape, which would mean that jaw movement does not affect the correlation between upper and lower tooth complexity. If tooth row shape is best explained by not only the opposing tooth row but also some measure of motion during occlusion, then tooth row shape is constrained by a suite of features and not just the opposing tooth row. Operating under the assumption that mandibular motion, as controlled by specific muscle action, is at least a partially heritable trait because of the limitations imposed by the organism’s musculoskeletal system this result would suggest integration of static and kinetic cranial features.

## Methods

### Specimens

16 species of Carnivora and 8 species of Dasyuromorphia were sampled, with a total of 34 specimens (20 and 14 specimens respectively; Table 1). Eight of these specimens were sampled previously [31] but were reanalyzed for this study. Specimens for this study were obtained from the Monash University Zoology Teaching Collection (MUZ), Museum Victoria (NMV), Finnish Museum of Natural History (FMNH), Swedish Museum of Natural History (SMNH), and the Museum für Naturkunde (ZMB). Specimens were chosen to reflect the taxonomic breadth of the two orders and for relatively unworn, complete upper and lower tooth rows. When possible, two specimens were sampled of each species.

**Table 1:** Specimens and measurements

**Order**	**Family**	***Genus***	***species***	**Collection**	**Number**	**upper tooth row length**	**lower tooth row length**	**upper OPCR**	**lower OPCR**	**t**	**d**	**a**	**w**
Carnivora	Canidae	Alopex	lagopus	FMNH	1345	3	3	154.25	96.75	3.22	8.71	22.21	56.20
Carnivora	Canidae	Canis	mesomelas	NMV	C32235	3	3	147.25	124.38	5.90	7.54	38.05	64.82
Carnivora	Canidae	Canis	aureus	ZMB	52447	3	3	172.62	132.88	3.37	10.16	19.73	70.00
Carnivora	Canidae	Vulpes	vulpes	NMV	C25076	3	3	153.38	111.38	4.44	9.75	24.49	65.48
Carnivora	Canidae	Vulpes	vulpes	NMV	C25077	3	3	150.12	113.62	4.10	8.43	25.93	66.88
Carnivora	Felidae	Acinonyx	jubatus	FMNH	U31	2	1	52.62	36.50	4.80	11.41	23.39	108.60
Carnivora	Felidae	Neofelis	nebulosa	NMV	R11997	2	1	42.62	18.38	4.71	14.04	18.55	97.48
Carnivora	Herpestidae	Herpestes	ichneumon	ZMB	83028	3	2	144.25	101.12	2.87	6.04	29.46	41.70
Carnivora	Herpestidae	Mungos	mungo	NMV	R1555	3	2	120.25	80.62	2.70	4.46	31.16	32.81
Carnivora	Herpestidae	Suricata	suricatta	NMV	R2454	3	2	162.62	64.12	1.12	1.24	42.18	38.63
Carnivora	Herpestidae	Suricata	suricatta	NMV	R2486	3	2	143.12	77.25	0.76	1.67	24.43	38.57
Carnivora	Hyaenidae	Crocuta	crocuta	FMNH	30.196	2	1	76.12	55.88	4.77	18.92	14.76	123.00
Carnivora	Mustelidae	Mustela	putorius	NMV	C22360	2	2	73.38	42.88	0.93	3.58	14.56	31.02
Carnivora	Mustelidae	Mustela	putorius	NMV	C32788	2	2	76.38	37.25	1.30	2.96	23.72	32.84
Carnivora	Mustelidae	Mustela	frenata	NMV	C11225	2	2	75.25	41.38	1.17	2.61	24.11	25.76
Carnivora	Mustelidae	Mustela	frenata	NMV	C31304	2	2	60.50	38.88	1.23	2.41	27.03	18.32
Carnivora	Mustelidae	Mustela	lutreola	ZMB	94308	2	2	119.25	71.25	0.92	3.31	16.36	34.70
Carnivora	Mustelidae	Vormela	peregusna	SMNH	A91 5107	2	2	113.25	56.75	0.94	2.94	19.05	28.90
Carnivora	Viverridae	Genetta	genetta	SMNH	A58 042	3	2	143.88	139.50	2.21	3.60	40.45	34.90
Carnivora	Viverridae	Viverra	zibetha	NMV	C1845	3	2	129.62	83.88	2.83	5.36	27.82	37.51
Dasyuromorphia	Dasyuridae	Dasycercus	cristicauda	NMV	C5364	4	4	251.88	188.25	1.34	1.80	36.59	22.65
Dasyuromorphia	Dasyuridae	Dasycercus	cristicauda	NMV	C5356	4	4	238.38	180.50	1.42	1.55	42.40	22.83
Dasyuromorphia	Dasyuridae	Dasyurus	geoffroii	NMV	C31515	4	4	212.12	170.62	2.03	3.67	28.93	38.99
Dasyuromorphia	Dasyuridae	Dasyurus	geoffroii	NMV	C31560	4	4	218.50	158.62	2.04	4.70	23.45	42.60
Dasyuromorphia	Dasyuridae	Dasyurus	maculatus	NMV	C6108	4	4	240.62	165.25	3.77	4.91	37.49	43.68
Dasyuromorphia	Dasyuridae	Dasyurus	maculatus	NMV	C29669	4	4	224.50	152.50	2.52	5.54	24.45	49.65
Dasyuromorphia	Dasyuridae	Dasyurus	hallucatus	MUZ	4735	4	4	214.88	162.25	2.43	3.30	36.38	31.62
Dasyuromorphia	Dasyuridae	Dasyurus	viverrinus	MUZ	5737	4	4	214.88	168.75	5.98	9.26	32.86	39.72
Dasyuromorphia	Dasyuridae	Phascogale	tapoatafa	NMV	C27059	4	4	292.88	204.88	1.84	2.27	38.99	28.73
Dasyuromorphia	Dasyuridae	Phascogale	tapoatafa	NMV	C34784	4	4	301.62	190.25	1.37	2.20	31.91	24.13
Dasyuromorphia	Dasyuridae	Sarcophilus	harrisii	NMV	C6232	4	4	169.75	114.62	4.83	11.01	23.69	83.50
Dasyuromorphia	Dasyuridae	Sarcophilus	harrisii	NMV	C6233	4	4	162.88	116.00	5.21	11.92	23.61	87.68
Dasyuromorphia	Thylacinidae	Thylacinus	cynocephalus	NMV	C5748	4	4	133.38	98.75	6.01	11.44	27.73	86.76
Dasyuromorphia	Thylacinidae	Thylacinus	cynocephalus	NMV	C5747	4	4	144.38	102.62	8.79	12.72	34.65	108.77

The number of teeth in upper and lower tooth rows is reported, along with OPCR values for each tooth row. Other measurements are lateral translation (*t*), ventral rotational distance (*d*), sagittal occlusal angle (*a*), and width of the skull measured as glenoid fossae width (*w*).

### Three-dimensional scanning

Specimens were scanned with a Laser Design DS 2025 3D scanner with a RPS-120 probe (Laser Design Inc., Minneapolis, MN) scanning at 620 nm wavelength. Upper and lower tooth rows from the same side of the jaw were scanned. Additionally, as in Evans and Fortelius [31], the articular surface of the squamosal bone (glenoid fossa) and dentary condyle were also scanned. The tooth row surfaces and dentary-squamosal joint are usually sufficient to determine occlusal path in carnivorous mammals [31,32]. Tooth and condyle surfaces were coated with a light layer of talc or ammonium chloride (NH_4_Cl) to aid scanning.

Depending on the size of the specimen, specimens were scanned at resolutions ranging from 50 μm to 10 μm. Tooth rows were saved as point cloud files, which were imported into Geomagic 12 (Geomagic Inc., North Carolina, USA 2010) and extraneous information was reduced using custom macros (available in Supplementary Information). Point clouds were aligned and then combined. Following this, each point cloud was transformed into a polygon surface at a triangle size greater than point cloud spacing by approximately 5 μm.

### Mandibular movement

To manipulate the surface reconstruction in three dimensions and simulate the chewing sequence, surface polygon files were exported as PLY (Stanford Triangle Format) files. The PLY files for the mandible and skull were then imported into Blender v. 2.5 (The Blender Foundation, 2011) where they were positioned as in life. The dentary condyle was placed in the glenoid fossa and the mandible was positioned with protocones of the upper molars placed in the talonid basins of the lower molars (i.e. centric occlusion). The mediolateral axis through the center of the dentary condyle acted as the center of rotation during chewing, remaining stationary in the sagittal plane. In life, synovial joint tissue is present between the bones, so the condyle was positioned with a small space between it and the glenoid fossa.

To simulate the process of occlusion, we used techniques described in Evans and Fortelius [31]. Included here is a brief description of this method. The skull was held in place while the mandible was dorsoventrally rotated and mediolaterally translated until the upper and lower teeth first touch. The mandible was continually rotated dorsally, with the teeth moving across each other until centric occlusion was again reached. As the mandible rotates dorsally the lower tooth row intersects the upper tooth row, so the jaw was medially translated until the two tooth rows were only tangentially in contact. An example reconstruction for *Sarcophilus harrisii* is included as Additional files 1, 2, and 3. As stated above, carnivorous mammals are ideal for this method of reconstruction because the precise occlusion of the upper and lower tooth rows and rotation is constrained to being only at the dentary-squamosal jaw joint. Because of this, mandibular motion is constrained in the anteroposterior and dorsoventral axes increasing the ease of reconstruction.

Four measures were taken for each specimen to quantify mandibular motion: distance of mediolateral translation by mandible (lateral translation, *t*), dorsoventral distance in the sagittal plane between centric occlusion and initial point of tooth-tooth contact measured from primary occluding tooth (ventral rotational distance, *d*), and the angle in degrees from the vertical in the sagittal plane between centric occlusion and initial point of tooth-tooth contact (sagittal occlusal angle) (*a*). Additionally, the distance between the lateral margins of the glenoid fossae was recorded as a measure of body size (glenoid fossae width, *w*).

Prior to analysis, lateral translation distance and ventral rotational distance were divided by glenoid fossae width, then natural log transformed. These ratios are measures of the relative amount of mandibular motion. Sagittal occlusal angle was also natural log transformed. These measures were compared between the two sampled orders for significant differences in medians using the non-parametric Mann–Whitney U test.

### Tooth shape

Tooth shape was measured as the number of discrete orientation patches on the teeth in a row (OPC). This measure provides a good estimate of the complexity of a morphological surface, which corresponds to the number of orientation-delimited functional surfaces [24]. For OPC calculation, the carnivoran tooth row was defined as P^4^/m_1_ and posterior, while for Dasyuromorphia the tooth row was defined as M^1^/m_1_ and posterior. For each specimen, the occlusal surfaces of the upper and lower tooth rows were isolated and saved as vertex files. These files were converted in Surfer for Windows (Golden Software, Inc., Colorado) and custom GIS software was used to measure the OPC value of the reconstructions (Surfer Manipulator [24]). Each tooth row was standardized to 50 data rows per tooth, so a row of 4 teeth was standardized to 200 data rows while a row of 1 tooth was standardized to 50 data rows. This down-sampling method allows for a more fair comparison between specimens and is in contrast to previous studies using OPC [24,29,30]. Additionally, to reduce the effect of tooth row orientation, the mean of OPC values from eight rotations of the three dimensional reconstructions at multiples of 5.625° was used [30]. This alternative measure is called OPCR.

OPCR measurements were natural log transformed prior to analysis. The relationship between upper tooth row ln(OPCR) and lower tooth row ln(OPCR) was determined using an ordinary least squares regression. Average tooth OPCR values were calculated as the tooth row OPCR value divided by the number of teeth in that tooth row, then natural log transformed.

### Model comparisons

Generalized least squares (GLS) models were constructed for two hypothesis groups: upper tooth row OPCR as a response to lower tooth row OPCR and measures of mandibular motion, and lower tooth row OPCR as a response to upper tooth row OPCR and measures of mandibular motion. GLS is an extension of ordinary least squares (OLS) estimation, but allows for correlation between the predictors, an assumption of OLS [33,34]. GLS models were fitted by maximum likelihood and with a Gaussian spatial correlation structure in cases of multiple predictors.

Five different models were constructed for both responses of upper and lower tooth row complexity. Each model represents a unique hypothesis of morphological factors controlling tooth shape: tooth row shape is only controlled by 1. opposing tooth row shape; 2. opposing tooth row shape and relative lateral translation (*rt*); 3. opposing tooth row and relative ventral rotational distance (*rd*); 4. opposing tooth row, relative lateral movement (*rt*) and relative ventral rotational distance (*rd*); or 5. opposing tooth row and sagittal occlusal angle (*a*). Sagittal occlusal angle is a tangent transform of the ratio between lateral translation and ventral rotational distance and gives an alternate measure of mandibular motion. All variables were natural log transformed prior to model fitting.

Models were compared using the second order Akaike’s Information Criterion (AICc) [35,36] which is recommended for routine use with small sample sizes [37]. AICc is an estimation of the distance of the model from reality and lower AICc value indicates that a model explains a greater amount of the response variance as possible without being overfit to the data. The number of parameters (*K*), log-likelihood, ΔAICc, and Akaike weights are reported. Akaike weight is an approximation of the selection probability of a model. A ΔAICc value of less than 2 indicates little to no difference in model performance from the best performing model, while values between 4 and 8 indicate moderate model support and values greater than 10 indicate no model support [37]. The sum of Akaike weights in rank order to equal to or greater than 0.95 represents an approximate 95% confidence set of best models [37].

All analysis was performed in the R statistical programming environment [38] using the MuMIn [39], and nlme [40] packages.

## Results and discussion

### Morphological measures

**Figure 2:**
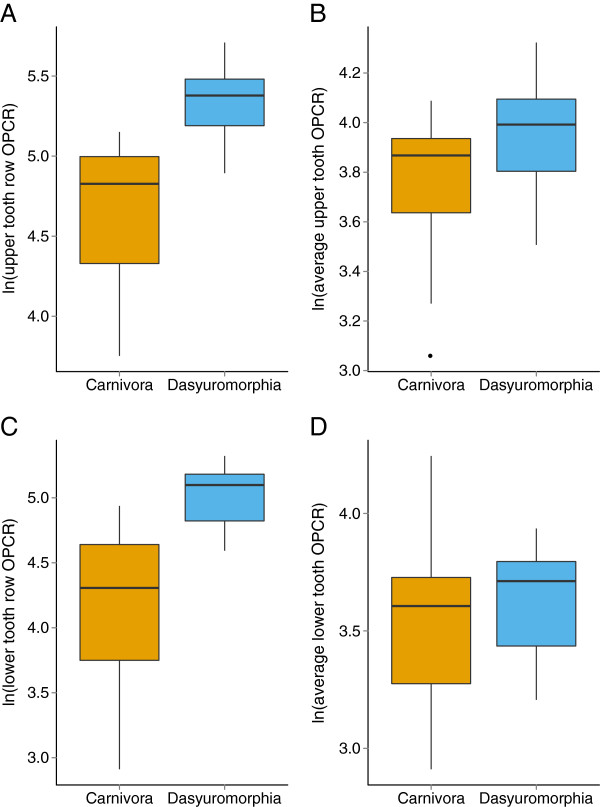
**Comparison of natural log transformed OPCR values for sampled Carnivora and Dasyuromorphia.** (**A**) Upper tooth row OPCR, (**B**) average upper tooth OPCR, (**C**) lower tooth row OPCR, (**D**) average lower tooth OPCR.

Carnivoran upper tooth row OPCR values range from 42.625 in *Neofelis nebulosa* to 172.625 in *Canis aureus* (Table 1). Dasyuromorph upper tooth row OPCR values range from 133.375 in *Thylacinus cynocephalus* to 301.625 in *Phascogale tapoatafa*. Lower tooth row variation is similar (Figure 2), with Carnivora ranging between 18.375 in *N. nebulosa* to 139.5 in *Genetta genetta* and Dasyuromorphia ranging between 98.75 in *T. cynocephalus* to 204.875 in *P. tapoatafa*. The OPCR value for the lower tooth row of *N. nebulosa*, a single molar, is among the lowest ever recorded [24,27-30].

Dasyuromorph upper tooth row OPCR values are significantly greater than carnivoran upper tooth row OPCR values based on a Mann–Whitney U test (p < 0.0001). This is also true for lower tooth row OPCR values (p < 0.0001). Dasyuromorph average upper OPCR values are significantly greater than carnivoran upper tooth OPCR values (Mann–Whitney U = 77, p < 0.05), while average lower tooth OPCR values are not significantly different (Mann–Whitney U = 104, p > 0.2). However, in the case of average upper OPCR values, *N. nebulosa* is an outlier with a very low average tooth OPCR. When this value is censored from analysis, dasyurid upper tooth OPCR values are only marginally significantly greater (Mann–Whitney U = 77, p ≈ 0.043). Additionally, there is great overlap between dasyuromorph and carnivoran average tooth OPCR values, both upper and lower (Figure 2). These results show that when tooth row length is taken into account, the two orders have similar dental complexity.

An OLS linear regression between upper OPCR as a response to lower OPCR in our samples reveals a strong and significant relationship (*r*^2^ = 0.92, p < 0.00001). Additionally when upper and lower tooth row OPCR values of previously sampled Carnivora [24] are plotted in the same space, the linear relationship between all of these upper tooth row OPCR response and lower tooth row OPCR values has a high correlation and is significant (r^2^ = 0.86, p < 0.00001). Additionally, parameter estimates of the slope and intercept for both linear models are within one standard error of each other. Of all 58 carnivoran and dasyurid specimens from this study and Evans et al. [24] only four have lower tooth row OPCR values that are greater than upper tooth row OPCR values.

Lateral translation distance varies between under a millimeter for *Suricata suricatta* to 8.79 mm for *T. cynocephalus*. There is a strong correlation between lateral translation and glenoid fossae width (r = 0.77). Dasyuromorph relative lateral translation distance is marginally significantly higher than that of carnivorans (Mann–Whitney U = 83, p ~ 0.047) though there is great overlap between the two orders, with Dasyuromorphia being almost entirely nested in the range of carnivoran values.

Similar to lateral translation distance, there is great variation in ventral rotational distance ranging from 1.236 mm for *S. suricatta* to 18.917 mm for *Crocuta crocuta.* Ventral rotational distance, as with lateral movement distance, is highly correlated with body size as measured by glenoid fossae width (r = 0.86). Ventral rotational distance shows a continuous distribution from smaller sized to larger sized taxa, and exhibits no taxonomic sorting. This is supported by the lack of significant difference in relative ventral rotational distance between Carnivora and Dasyuromorphia (Mann–Whitney U = 171, p > 0.2).

Sagittal occlusal angles range from 14.55° for *Mustela putorius* to 42.4° for *Dasycercus cristicauda.* Dasyuromorph sagittal occlusal angles are significantly greater than carnivoran sagittal occlusal angles (Mann–Whitney U = 76, p < 0.05); however, there is a large amounts of overlap between the two orders and dasyuromorphs have a narrower range of values.

Because of the large degree of overlap in all morphological measures, instead of considering these two orders as distinct, we will consider Carnivora and Dasyuromorphia as part of the same morphological continuum. When there are few subpopulations, small sample size, and large overlap in value means, linear mixed-effects models, which take subpopulation effects into account, are numerically equivalent to the simpler generalized least-squares method. Preliminary comparisons made between mixed-effects and GLS models showed they were numerically equivalent with no major difference in likelihood, indicating no major subpopulation effects. These results are consistent with traditional morphometric and geometric morphometric analyses of skull shape in carnivorous mammals suggest that Dasyuromorphia and Carnivora are part of a continuum of carnivorous skull shapes [41-43]. Additionally, our finding that dasyuromorphs occupy a much smaller range of morphologies than carnivorans is also consistent with previous studies [41,42,44,45].

This is the first study to measure dasyuromorph, and marsupial in general, OPCR values and we find that OPCR values in Carnivora and Dasyuromorphia occupy an overlapping range of values. Tooth row OPCR values are larger in dasyuromorphs than carnivorans, although average tooth complexities are nearly identical. The marginally greater median average tooth OPCR in dasyurids than carnivorans may be a product of the sampled carnivoran diversity. This also applies to our measures of mandibular motion where the marginally significant greater relative lateral translation in dasyurids than carnivorans may be a product of sampling. Increased sampling in Carnivora may also negate these findings. Current carnivoran sampling does not include more omnivorous or herbivorous species, such as bears. Jaw movement in bears is not as constrained by tooth shape as carnivorans with more blade-like teeth and our methodological focus on centric occlusion cannot be applied in less constrained systems.

### Model comparisons

The best performing model of upper tooth row OPCR is a combination of lower tooth row OPCR and relative ventral rotational distance (Table 2). This five parameter linear model had a log-likelihood of 27.733, an AICc score of −43.3, and an Akaike weight of 0.757. The second best model is the most complicated model of lower tooth row OPCR, relative lateral movement and relative ventral rotational distance with an Akaike weight of 0.192, and the ΔAICc between the first and second models of 2.75 indicates marginal difference between these models. The third best model is lower tooth row OPCR and relative lateral movement with an Akaike weight of 0.046 and ΔAICc of 5.61, again indicating marginal difference between this model and the best model. Both the lower tooth row OPCR alone model and lower tooth row OPCR and sagittal occlusal angle models are poorly performing (Table 2), indicating much lower empirical support for these models. A simple 95% confidence set is almost achieved by summing the Akaike weights of the best two AICc models at 0.949, and inclusion of the third AICc best model increases the total sum to 0.995.

**Table 2:** Model selection results for upper tooth row as response

**Model No.**	**Intercept**	**lower OPCR**	***rt***	***rd***	***a***	***df***	**logLik**	**AICc**	**ΔAICc**	**Akaike weight**
3	0.98	0.77		−0.21		5	27.73	−43.32	0.00	0.757
4	0.87	0.78	−0.04	−0.18		6	27.84	−40.58	2.75	0.192
2	0.76	0.81	−0.17			5	24.93	−37.71	5.61	0.046
1	1.37	0.79				3	19.43	−32.06	11.26	0.003
5	1.36	0.74			0.07	5	22.03	−31.92	11.41	0.003

Models are presented in order of relative best to worst. Shown are parameter estimates, number of parameters (*df*), log-likelihood, AICc, ΔAICc, and Akaike weight for each model.

The best model of lower tooth row complexity is upper tooth row OPCR and relative ventral rotational distance with a log-likelihood of 20.214, AICc of −28.3, and Akaike weight of 0.446 (Table 3). The second best model is upper tooth row OPCR and relative lateral translation distance with an Akaike weight of 0.276 and with a ΔAICc between the first and second models of 0.96 meaning there is no discernible difference between the two models. The third best model of lower tooth row is the complex model of upper tooth row OPCR, relative lateral movement, and relative ventral rotational distance with an Akaike weight of 0.273 and a ΔAICc of 0.99 indicating no discernible difference between this model and the first two [37]. Both the lower tooth row OPCR alone model and lower tooth row OPCR and sagittal occlusal angle indicates little empirical support for these models (Table 3). A 95% confidence set of selected models requires only the top three AICc best models, with a sum of Akaike weights of 0.995.

**Table 3:** Model selection results for lower tooth row as response

**Model No.**	**Intercept**	**upper OPCR**	***rt***	***rd***	***a***	***df***	**logLik**	**AICc**	**ΔAICc**	**Akaike weight**
3	−0.86	1.21		0.25		5	20.21	−28.29	0.00	0.446
2	−0.26	1.11	0.24			5	19.73	−27.33	0.96	0.276
4	−0.47	1.16	0.13	0.16		6	21.21	−27.30	0.99	0.273
1	−1.23	1.17				3	12.78	−18.76	9.53	0.004
5	−1.34	1.16			0.04	5	14.48	−16.82	11.47	0.001

Models are presented in order of relative best to worst. Shown are parameter estimates, number of parameters (*df*), log-likelihood, AICc, ΔAICc, and Akaike weight for each model.

For both upper and lower tooth row OPCR as model responses, the numerically best performing model was the opposing tooth row OPCR and relative ventral rotational distance. However, in both cases these models do not greatly outperform the next two best models. We recommend the use of multimodel inference methodology, such as weighted parameter averaging, to take this uncertainty in parameter estimates and variance into account when making estimates from these models [37,46].

For upper tooth row OPCR as response, our 95% confidence set is made up of our three best performing models. The only variables in these models are lower tooth row OPCR, relative ventral rotational distance and relative lateral translation of the mandible. Multi-model inference and parameter averaging would be best limited to just the best three models. Inclusion of the last two models in model averaging is most likely unnecessary, as they have large ΔAICc values and low Akaike weights. From the three model confidence set we find that upper tooth row OPCR decreases if lower OPCR is held constant and one of relative ventral rotational distance or relative lateral translation increases and the other is held constant (Table 2). If the lower OPCR variable increases and both ventral rotational distance and relative lateral translation are held constant, the upper OPCR increases.

There are similar results for lower tooth row OPCR as a response. The parameters in the three models of our 95% confidence set are upper tooth row OPCR, relative ventral rotational distance and relative lateral translation of the mandible. The second and third best performing models have ΔAICc values of less than 1, indicating these models are virtually identical is explaining lower tooth row OPCR. As such, the best model would be made weighted averaging of the estimated parameters of these three models. Inclusion of the final two models is unnecessary, as they both have high ΔAICc values and low Akaike weights. From the confidence set of these three models we find that lower tooth row OPCR increases if upper OPCR is held constant and one of relative ventral rotational distance or relative lateral translation increases and the other is held constant (Table 3). Similar to models with upper OPCR response, if the upper OPCR variable increases and both ventral rotational distance and relative lateral translation are held constant, lower OPCR increases.

In both model selection cases, models with just OPCR or with OPCR and sagittal occlusal angle as predictors are the two worst performing models, by moderate difference in AICc values. The poor relative model support of OPCR as the sole predictor means that the best possible inference of the opposing tooth row OPCR should not be based entirely from OPCR values. Instead more complex models are advisable (see below). The poor performance of OPCR and sagittal occlusal angle is unexpected, as this value represents the angle of movement of the jaw during closing. While this angle measure is strongly correlated with both upper and lower OPCR, the use of GLS with a Gaussian spatial correlation to control for this multicollinearity leads to low likelihood models which perform worse than models including the linear measures.

## Conclusions

The combination of kinetic and static measures has not previously been used in the context of integration, though represents an important part of studies of comparative anatomy [47]. The use of three dimensional reconstructions allows for measurements, such as ventral rotational distance, to be more easily measured than other less exact or time-intensive techniques such as estimating occlusal direction from microwear or x-ray cineradiography [48-54]. Our results indicate that the inclusion of kinetic measures is valid in statistical models of morphological integration. The inclusion of kinetic measures in integration studies provides an enhanced understanding of the complex interplay of features and processes constraining morphology. While the effects of the measures of mandibular motion are quite small, their inclusion increases the explanation of the variance in the response for our different models without overfitting our data, meaning that there is an aspect of tooth row shape not explained by just the shape of the opposing tooth row. It is important to note, however, that the degree of these changes should be calculated through model averaging methods [37,46]. Morphology, especially ecologically-critical morphology, has functional requirements, thus it makes sense that the best performing models of tooth row shape include both kinetic-type in addition to static-type measures.

Additionally, the use of information theoretic model selection criteria also provides a method for quantifying the uncertainty between different hypotheses of constraint. Model selection uncertainty allows for better parameter and variance estimates by using weighted averages of values from the set of best models [37,46]. Also, by increasing sample size, more complex models with possible tapering effects, which are only observable at large sample sizes, can be compared. Currently, this is beyond the reach of this data set. A potential source of new taxa would be Creodonta, an extinct group of carnivorous mammals with lower molar structure similar to dasyurids. The inclusion of a wider range of carnivorous mammals outside Carnivora and Dasyuromorphia would increase the generality of this analysis.

Future analysis may wish to consider the interaction of relative lateral translation distance and relative ventral rotational distance as a predictor instead of the solely additive relationship between these two variables. This new variable is an alternative relation between the horizontal and vertical movement and may allow for a better understanding of tooth row shape. Preliminary exploratory post hoc analysis indicates the inclusion of this variable may be unnecessary, though increased sample size may recover possible tapering effects.

In conclusion, our results provide quantitative support of long-standing hypotheses of tooth row shape as being influenced by mandibular motion in addition to the opposing tooth row [25,26]. Our results show that, even at low sample sizes, increasing model complexity for estimating tooth row OPCR values by including measures of mandibular motion is warranted, produces a better model in terms of AICc, and that the relative importance of these measures should be taken into account during analysis.

## Competing interests

The authors declare that they have no competing interests.

## Authors’ contributions

PDS conceived the study. PDS and ARE gathered and scanned specimens. PDS measured mandibular motion. PDS conducted statistical analysis with input from ARE. PDS wrote the manuscript with input from ARE. All authors read and approved the final manuscript.

## Supplementary Material

Additional file 1 S_harrisii_circle.avi avi [http://www.videolan.org/vlc/] Reconstructed occlusion of *Sarcophilus harrisii.* Movie of three-dimensionally reconstructed occlusion in *Sarcophilus harrisii*. Upper tooth row and skull are yellow, lower tooth row and mandible are blue. Click here for file

Additional file 2 S_harrisii_condyle.avi avi [http://www.videolan.org/vlc/] Dentary condyle of *Sarcophilus harrisii* during occlusion. Movie of lateral movement of dentary condyle during occlusion in *Sarcophilus harrisii*. Skull is yellow, mandible is blue. Click here for file

Additional file 3 S_harrisii_teeth.avi avi [http://www.videolan.org/vlc/] *Sarcophilus harrisii* teeth during occlusion. Three-dimensionally reconstructed occlusion in *Sarcophilus harrisii.* Upper tooth row and skull are yellow, lower tooth row and mandible are blue. Click here for file
